# Transferring control demands across incidental learning tasks – stronger sequence usage in serial reaction task after shortcut option in letter string checking

**DOI:** 10.3389/fpsyg.2014.01388

**Published:** 2014-11-28

**Authors:** Robert Gaschler, Julian N. Marewski, Dorit Wenke, Peter A. Frensch

**Affiliations:** ^1^Interdisciplinary Laboratory Image Knowledge Gestaltung, Humboldt-UniversitätBerlin, Germany; ^2^Department of Psychology, Universität Koblenz-LandauLandau, Germany; ^3^University of LausanneLausanne, Switzerland; ^4^Max Planck Institute for Human DevelopmentBerlin, Germany; ^5^Department of Psychology, Humboldt-UniversitätBerlin, Germany

**Keywords:** incidental learning, information reduction, serial reaction task, transfer, cognitive conflict, instruction following, pliance

## Abstract

After incidentally learning about a hidden regularity, participants can either continue to solve the task as instructed or, alternatively, apply a shortcut. Past research suggests that the amount of conflict implied by adopting a shortcut seems to bias the decision for vs. against continuing instruction-coherent task processing. We explored whether this decision might transfer from one incidental learning task to the next. Theories that conceptualize strategy change in incidental learning as a learning-plus-decision phenomenon suggest that high demands to adhere to instruction-coherent task processing in Task 1 will impede shortcut usage in Task 2, whereas low control demands will foster it. We sequentially applied two established incidental learning tasks differing in stimuli, responses and hidden regularity (the alphabet verification task followed by the serial reaction task, SRT). While some participants experienced a complete redundancy in the task material of the alphabet verification task (low demands to adhere to instructions), for others the redundancy was only partial. Thus, shortcut application would have led to errors (high demands to follow instructions). The low control demand condition showed the strongest usage of the fixed and repeating sequence of responses in the SRT. The transfer results are in line with the learning-plus-decision view of strategy change in incidental learning, rather than with resource theories of self-control.

## INTRODUCTION

**FIGURE 1:**
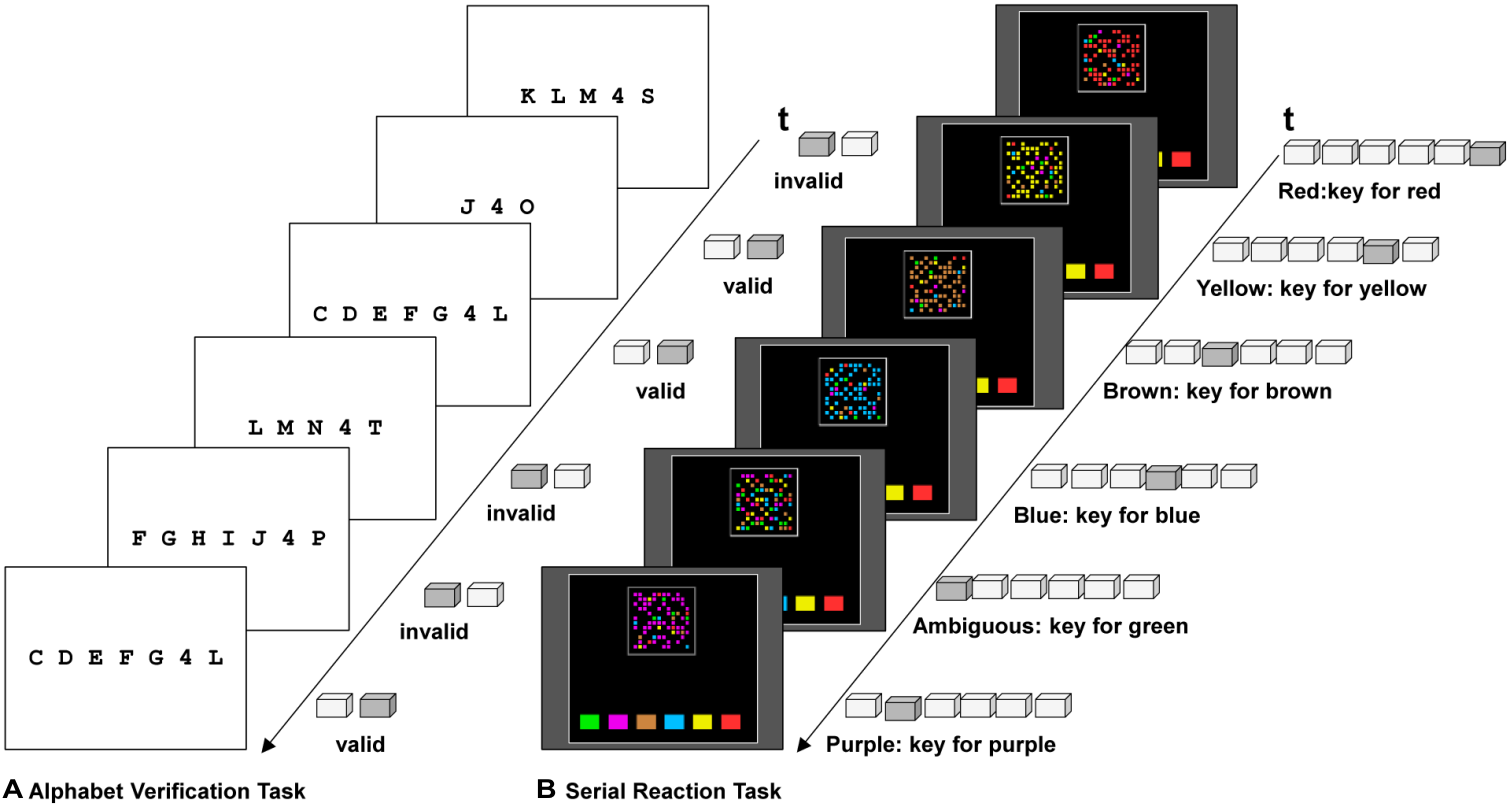
**Task material in the alphabet verification task **(A)** and the serial reaction task (SRT; **B**)**.

The human factors literature counts many cases where, with experience, people change from processing a task as instructed to applying a shortcut (Reason, 1990; Niessen et al., 1999; Underwood et al., 2002). This has triggered experimental work on incidental learning to explore the role of cognitive control in strategy change (e.g., Strayer and Kramer, 1994; Haider and Frensch, 1999; Touron and Hertzog, 2004a,b; Haider et al., 2005; Hoyndorf and Haider, 2009). In some experimental setups participants who had discovered a shortcut were faced with high vs. low demands to adhere to instruction-coherent task processing instead of applying the shortcut. For instance, Gaschler and Frensch (2009) instructed participants to check strings for alphabet errors (see **Figure 1A** for an example). With practice, participants could learn that some string positions rarely contained alphabet errors so that time could be saved by skipping these positions when checking the strings. Experimental conditions differed in the amount of alphabet errors in these less relevant string positions. Disregarding the instruction to exhaustively check the strings led to few errors for one group of participants (low demand to secure adherence to instructions). On average this group showed a higher rate of shortcut usage than the group for which more errors would have resulted from disregarding the instructions (high control demand).

Importantly, the number of errors that one would commit using the shortcut seemed to affect performance by influencing the probability that a participant fully used the shortcut vs. refrained from using it. Thus, an all-or-non adjustment of control was observed. While some participants started to use the shortcut on all following trials after some practice, others completely refrained from using it. Conflict level (i.e., level of errors implied by shortcut usage) was influencing how many of the participants used the shortcut, rather than to what extent they used it. The link between conflict level and shortcut-based errors seems plausible, given that response errors have been tied to similar control processes and neural substrates driving behavioral adjustment as the ones involved in case of competing response tendencies, decision uncertainty and unfavorable outcomes (e.g., Ridderinkhof et al., 2004). The adjustment of shortcut usage to control demands is in line with work suggesting that strategy change in incidental learning is based on a general decision to apply or not apply an incidentally discovered shortcut (e.g., Haider and Frensch, 1996, 2002). When people apply the shortcut, they do so for practiced and novel stimuli alike (cf. Gaschler et al., 2014a). For instance, Touron and Hertzog (2004a,b) reported that most older (as compared to younger) research participants in incidental learning experiments were reluctant to apply a shortcut they had learned. While they had sufficiently memorized the set of search items in a match-to-sample visual search task to avoid visual search in favor of faster memory search, they continued to solve the task as instructed. As the shortcut option is not mentioned in the instructions of incidental learning tasks, participants cannot be sure that the shortcut option they eventually discovered will hold throughout the experiment. In addition to the insecurity regarding the reliability of the shortcut, some participants reported reluctance to apply a shortcut because they placed more emphasis on accuracy than speed (see also Haider and Frensch, 1999).

Following or disregarding instructions is not a one-shot game. Learning theorists have suggested that people follow instructions, because they have generalized episodes in which instruction following was reinforced (e.g., Hayes et al., 1986, 2001; Törneke et al., 2008). On the one hand, participants might thus learn about contexts in which it generally pays off to follow instructions. On the other hand, they should also be able to learn under which conditions it is more advantageous to apply a shortcut instead of sticking to instruction-coherent task processing. As suggested above, people might decide to apply a shortcut, based on the experience that it rarely or never leads to errors. However, adaptation to the conflict level that a shortcut implies might not only affect processing of the current task, but also transfers to other tasks. Having experienced an incidental learning task in which a potential shortcut leads to few vs. many errors might influence the likelihood to adopt a shortcut discovered in a later task.

Theories that view strategy change as a phenomenon involving both, the learning of a shortcut option, as well as the decision to apply it or to refrain from applying it (e.g., Touron and Hertzog, 2004a,b; Gaschler et al., 2014a) can predict that experience with one incidental learning task offering a shortcut option, transfers to a second incidental learning task (see discussion for competing theories). Prior experience with low demands to refrain from shortcut usage will foster shortcut usage in the next incidental learning task. This is because the experience that shortcut application did not lead to errors in the first task, could bias the expectation that this would not be the case either in the next task (for expectation effects on conflict processing within task cf. Duthoo et al., 2013; or review by Gaschler et al., 2014b). Thus, after working on a task in which a shortcut could be discovered and adopted, participants should be more likely to use a shortcut on a task presented later on. Conversely, prior experience with a setup where the demands to refrain from applying a shortcut are high, could lead to the expectation of high control demands for the next task. In this case, participants would be more likely to refrain from using a shortcut in Task 2. A baseline condition not working on Task 1 should show intermediate levels of shortcut usage.

In the current experiment, we combined two established incidental learning tasks in order to study transfer of control demands. We used control demands in the task applied first as an independent variable and performance in the second task as a dependent variable. In two conditions participants first worked on the *alphabet verification task* (e.g., Haider and Frensch, 1996; Green and Wright, 2003; **Figure 1A**) and then on a variant of the *serial reaction task* (SRT; e.g., Nissen and Bullemer, 1987; Abrahamse et al., 2010; **Figure 1B**). In the alphabet verification task participants are instructed to tediously check alphanumeric strings. Yet they learn that these strings contain a redundant section that could be skipped. In the SRT participants receive choice reaction instructions for a consistent stimulus-response mapping. Instead of choosing reactions based on the stimulus of the current trial as instructed, they can substantially simplify task processing by learning and applying the fixed repeating sequence of stimuli and responses. While in typical variants of the SRT the sequence is long and learning remains implicit (e.g., Abrahamse et al., 2010), we used a variant with a short and simple sequence – similar to experiments in which participants have become aware of the sequence and became able to produce reactions without paying attention to the stimuli (cf. Haider and Rose, 2007; Rünger and Frensch, 2008; Schwager et al., 2012). Our variant of the SRT was constructed such that large gains in performance based on sequence knowledge were possible. Tubau et al. (2007, see also Verwey and Wright, 2014) showed that sequence knowledge allows participants to change from stimulus-based responding to memory-based responding. We used a rather simple repeating sequence. The six stimuli and keys were each presented once. The rationale behind this setup, established in Rünger and Frensch (2008), is that people would neither find it difficult to represent nor implement the shortcut option, once they have learned it – allowing us to focus on control demands (minimizing strategy performance problems).

As a novel approach to continuously assess sequence knowledge throughout practice, we included randomly interspersed ambiguous stimuli. If participants know the repeating sequence, they can give the response that would have been due according to the fixed repeating sequence if an ambiguous stimulus is presented. Otherwise they have to guess a response as the stimulus cannot be discriminated. In addition, we adopted a more traditional measure of sequence knowledge. Studies using the SRT usually measure sequence knowledge after practice with the sequential regularity by assessing the reaction time slowing in off-sequence blocks or randomly interspersed off-sequence deviant trials in comparison to trials following the sequence (e.g., Schvaneveldt and Gomez, 1998; Shanks et al., 2003; Abrahamse et al., 2010; Gaschler et al., 2012). We used this measure by randomly inserting deviant trials. We did so only at the end of practice, as reports of participants starting to rely on memory-based instead of stimulus-based response selection in the SRT come from setups using sequences without deviants (e.g., Tubau et al., 2007; Rünger and Frensch, 2008; Schwager et al., 2012). Providing a further reason for saving this measure for the end of practice, Verwey and Wright (2014) reported RT data suggesting that deviants might suppress the expression of sequence knowledge.

In summary, the present study set out to examine whether shortcut usage in one task transfers to a subsequent task. We hypothesized that prior experience with a setup where a shortcut can be safely applied should lead to increased shortcut usage in a second incidental learning task. Conversely, prior exposure to a shortcut which would lead to errors should reduce shortcut usage in the second task.

## MATERIALS AND METHODS

### PARTICIPANTS

One hundred and four students from different Berlin-based universities took part in the experiment and were paid € 10 (69 female; mean age 24.8 years, SD = 5.2). When entering the lab, participants were randomly assigned to the low or high control demand condition without knowledge of the experimenter. Conditions differed in the variant of the alphabet verification task that they were presented before working on the SRT. The participants of the baseline condition worked only on the SRT. Therefore, they were in the lab for a shorter time and were treated separately by the experimenter. Exclusion of four participants (see results) led to 32 participants in both, the high and the low control demand condition and 36 participants in the baseline condition. The experiment took place in the laboratories of the Psychology Department of Humboldt-Universität Berlin. We obtained informed consent from the participants and approval by the ethical review board.

### MATERIALS AND APPARATUS OF TASK 1: ALPHABET VERIFICATION TASK

The stimuli in the alphabet verification task consisted of 48 alphanumeric strings (e.g., C D E F G 4 L; see **Figure 1A**), presented two times in each of the four blocks of practice. Half of the strings were *valid*, following the order of the alphabet; the other half were *invalid*, deviating from it. The digit 4 in the letter-digit-letter triplet indicated that the next four consecutive letters of the alphabet needed to be skipped at this string location, and that the string would continue with the fifth letter. Thus, “M 4 R,” for instance, was to be interpreted as “M, skip N, O, P, Q, continue with R.” There were either no, two or four letters forming a prefix before the letter-digit-letter triplet.

In the low control demand condition, violations of the alphabetical order only occurred in the letter-digit-letter triplet (five instead of the indicated four letters fitting the void). The prefix (i.e., the letter in front of the letter-digit-letter triplet) was always correct. In the high control demand condition, however, the prefix was free of errors only in 75% rather than 100% of the trials. The letters outside the triplet could therefore not be safely ignored. As in other work on strategy change with the alphabet verification task (e.g., Gaschler and Frensch, 2007), the length of the prefix was varied in order to obtain a reaction time measure of the extent of prefix processing. As long as participants adhere to the instructions and check the strings exhaustively, longer strings should lead to higher reaction times as compared to shorter strings. The impact of string length on RT should diminish with practice to the extent that participants stop to check the prefixes.

Each trial started with a fixation cross presented centrally for 200 ms that was followed by an alphanumeric string. Strings were centrally presented in bold Courier New font, size 26, at the center of a 17-inch CRT screen in black color on a light yellow background, controlled by a PC. The font ensured constant spacing between letters. The letters were ∼1.1° × 0.9° in size. Consecutive letters appeared ∼0.9° apart on the screen. After the manual response was registered, the string was erased from the screen and there was a blank interval of 200 ms before the fixation cross of the next trial appeared. Incorrect responses were immediately followed by a high tone as error signal. Participants responded by pressing either the “y” or the “,” key on the second row from the bottom on a standard German PC keyboard. Half of the participants were instructed to use the “y” key to indicate that a string was valid and the “,” key to indicate that the string was invalid; for the other half, the key assignment was reversed.

In the computerized instructions, the characteristics of the alphanumeric strings were described, and participants were shown how to evaluate the strings. Participants were instructed to pay attention to the entire string because errors could occur anywhere in the string. Furthermore, they were told to respond as quickly as possible while keeping the rate of errors below 10%. The alphanumeric strings used as examples in the instructions and in the 10 practice trials (triplets starting with E and F) contained violations of the alphabetical order outside the letter-digit-letter triplet and were not from the pool of material used for the rest of the task. The task was completed within ∼45 min.

### MATERIALS AND APPARATUS OF TASK 2: SRT

In each trial, participants saw a random cloud of 72 dots, each colored in one of six colors for 250 ms in a centrally presented frame (**Figure 1B**). The frame was drawn in gray lines on a black background. Afterward, the cloud disappeared and the program awaited a response. A new stimulus was displayed after a response stimulus interval (RSI) of 100 ms. In order to allow for execution of fast response sequences (e.g., based on sequence knowledge), the stimulus presentation ended early in case participants responded during the stimulus presentation. Except on some irregular trials, (see below) one color was much more frequent (52 dots) as compared to the other five (four dots each) in each trial. At a distance of 3.6° beneath the 4.4° × 4.4° frame with the dots, a row of colored squares indicated the mapping between the dominant color and the response. Each color box was 1.2° high and 1° wide and the spacing was 0.5°. Participants responded with the keys on the lower row of the keyboard (X to M) using index, middle, and ring fingers which they should keep resting on the keys. The keys were numbered 1 to 6 with stickers and the mapping of colors to keys was constant throughout the task.

Unbeknownst to the participants, stimuli (dominant stimulus colors) and the required responses followed a simple fixed repeating sequence. Sequences were drawn from a pool of 24 first-order sequences of length six. Each of the six stimuli and responses occurred once and this sequence was constantly repeated. To avoid salient spatial patterns in the response positions, the sequences did not contain “runs” of three or more adjacent response locations (e.g., 1-2-3, 6-5-4 with responses numbered from left to right; cf. Rünger and Frensch, 2008). The selection of the sequences was matched between the conditions of the experiment.

Each of the seven blocks of the SRT consisted of 108 regular and 12 irregular trials. An auditory error feedback was presented during the RSI on regular trials, while any response in the irregular trials was regarded as correct. With (1) ambiguous and (2) deviant trials, we used two different kinds of irregular trials in different blocks in order to assess usage of sequence knowledge – the dependent variable of the experiment. Stimuli in irregular trials in Blocks 1 to 6 were maximally ambiguous. The cloud of dots contained dots of all colors with equal frequency. Thus, the stimulus did *not* suggest any of the six responses more strongly than the other five. Therefore, sequence knowledge should be measurable in a response bias. For instance, the ambiguous stimulus in **Figure 1B** elicits the response for green. As this response was due according to the fixed repeating sequence, such a response suggests sequence knowledge. No sequence knowledge would be evident if the participant pressed the key according to the sequence only at chance level (match in 1/6th of the ambiguous trials). If participants acquire knowledge about the fixed repeating sequence and decide to exploit it for the simplification of task processing, they should not only pass chance level in choosing responses according to the repeating sequence. Rather they should start to consistently respond according to the fixed and repeating sequence in the ambiguous trials.

In Block 7, the stimuli in deviant trials had a dominant color that did not follow the sequence. For instance, instead of a cloud of predominantly yellow dots that should appear based on the repeating sequence, a predominantly blue stimulus might be randomly inserted instead. Random deviants were drawn such that immediate repetitions of responses were avoided. Sequence knowledge was assessed as the reaction time difference between, on the one hand, the irregular trials and their immediate successors, and, on the other hand, the remainder of the trials with correct responses. We included the immediate successor of the deviant as a potentially slowed trial in order to increase the number of trials available for the RT estimate.

### PROCEDURE

Except for the baseline condition, participants started the experiment with the alphabet verification task. No references were made as to whether a part of the stimuli could be safely ignored or not. After completing the alphabet verification task, the experimenter started the automatized instructions of the serial reaction time task. Participants were told that this task is a speeded forced choice stimulus discrimination task. In doing so, no underlying regularities in the task material were mentioned. The experimenter then watched the first five trials to make sure that participants had properly understood the instructions. Only after completing the SRT participants were asked whether or not (forced choice) it would have been possible for them to skip checking a part of the string positions of the alphabet verification task (see results on manipulation check). Also the experimenter inquired about verbalizable sequence knowledge (SRT). Participants were asked to recall the fixed repeating sequence or otherwise guess a sequence of six elements. For each participant, the pattern of the correctly verbalized portion(s) of the trained sequence was compared to a simulation in order to estimate the likelihood that it was based on guessing (see Rünger and Frensch, 2008). The simulation determined how often the specific pattern of correct verbalizations observed for a participant (e.g., a triplet correct) would be obtained by matching the training sequence with a randomly generated sequence 10 million times. If the specific pattern of correct verbalizations occurred with low relative frequency in random matching, it was likely not the result of guessing.

## RESULTS

### SCREENING OF THE DATA

Screening of the data suggested that there was no speed–accuracy trade-off. In both tasks error trials tended to be slower rather than faster as compared to correct trials. In the low control demand condition, one participant did not fully complete the alphabet verification task and three participants were excluded because of error rates higher than 30%. The mean error rate of the remaining participants of the high control demand condition (*N* = 32) and those of the low control demand condition (*N* = 32) was 7.5% for either group. See below for SRT error rates of these conditions and the baseline condition (*N* = 36).

### MANIPULATION CHECKS

**FIGURE 2:**
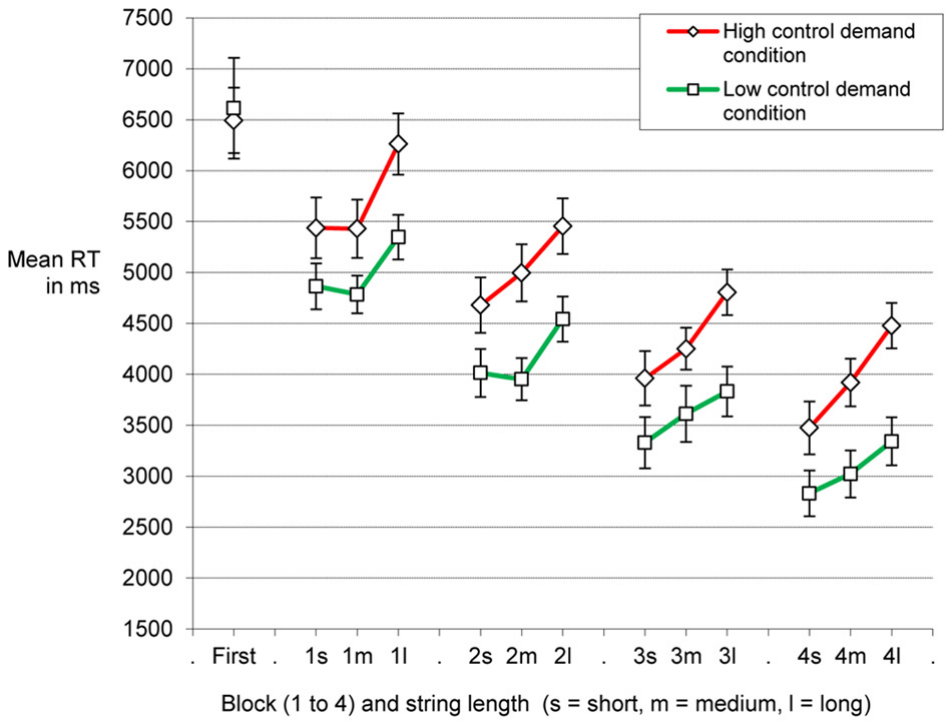
**Means of individual median reaction times.** On the *x*-axis, reaction times for short, medium, and long strings (s, m, l) are grouped together by block in order to display the amount of processing of irrelevant information. The impact of string length on RT is stronger in the high control demand condition as compared to the low control demand condition, indicating more processing of the additional letters when information reduction is not possible. Note that error trials as well as RT of trials with violations of the alphabetical order (high control demand condition) were excluded. Note that performance for the two conditions was very similar during the very first trials of Block 1 and quickly diverged afterward. This was evident when we compared (a) the trials of Block 1 that occurred before participants in the high control demand condition encountered the first strings with violation of the alphabetical order outside the letter-digit-letter triplet with (b) the yoked trials of the low control demand condition. As soon as participants in the high control demand condition were confronted with the strings including incorrect prefixes (RT from the latter trials being excluded from main analysis) RT differences between the conditions quickly developed. Error bars: between-subjects standard error of the mean.

In the main analysis below we employed presence and variant of the alphabet verification task (high control demand condition, low control demand condition, baseline condition) as an independent variable for performance in the SRT. Beforehand, we checked whether the manipulation of the feasibility of information reduction actually led to performance effects in the alphabet verification task itself. As participants in the low control demand condition could safely skip to check some of the string positions, it was to be expected that they should be generally faster than participants of the high control demand condition. Furthermore, RTs in the low control demand condition should be less strongly influenced by string length, because the string prefixes (letters before the letter-digit-letter triplet) of varying length did not contain to-be-spotted alphabet errors and thus could be skipped. The data presented in **Figure 2** are in line with these predictions. A mixed ANOVA on RTs, including block and string length as within-subjects factor and control demand as a between-subjects factor showed a main effect of control demand condition, *F*(1,62) = 7.53, MSE = 16480000, *p* = 0.008, $\eta_{p}^{2}$ = 0.11, of block of practice, *F*(3,186) = 76.93, MSE = 1601747, *p* < 0.001, $\eta_{p}^{2}$ = 0.55, and of string length on RT, *F*(2,124) = 72.43, MSE = 447654, *p* < 0.001, $\eta_{p}^{2}$ = 0.54. Furthermore, there was an interaction between control demand condition and string length, *F*(2,124) = 4.53, MSE = 447654, *p* = 0.013, $\eta_{p}^{2}$ = 0.07, as string length was of less influence for participants of the low control demand condition than for participants who could not safely skip to check the string prefixes. With practice there was a decrease of processing of the string positions containing alphabet errors rarely or never, *F*(6,372) = 2.55, MSE = 204073, *p* = 0.02, $\eta_{p}^{2}$ = 0.04, for the interaction between block of practice and string length (other *F*s < 1.1). Note that we applied Greenhouse–Geisser correction in the ANOVAs when necessary.

Participants proved knowledgeable about whether or not information reduction had been possible in the version of the alphabet verification task they had been practicing. When asked to guess whether or not the prefix letters in their version of the task had or had not always been in correct alphabetical order, all participants of the high control demand condition correctly stated that errors in the alphabetical order had occurred in the letters placed in front of the letter-digit-letter triplet. Four of the participants in the low control demand condition incorrectly stated that this was the case in their version of the task as well, while the others correctly reported that the prefixes had always been correct.

### SLOWING BY OFF-SEQUENCE STIMULI

**FIGURE 3:**
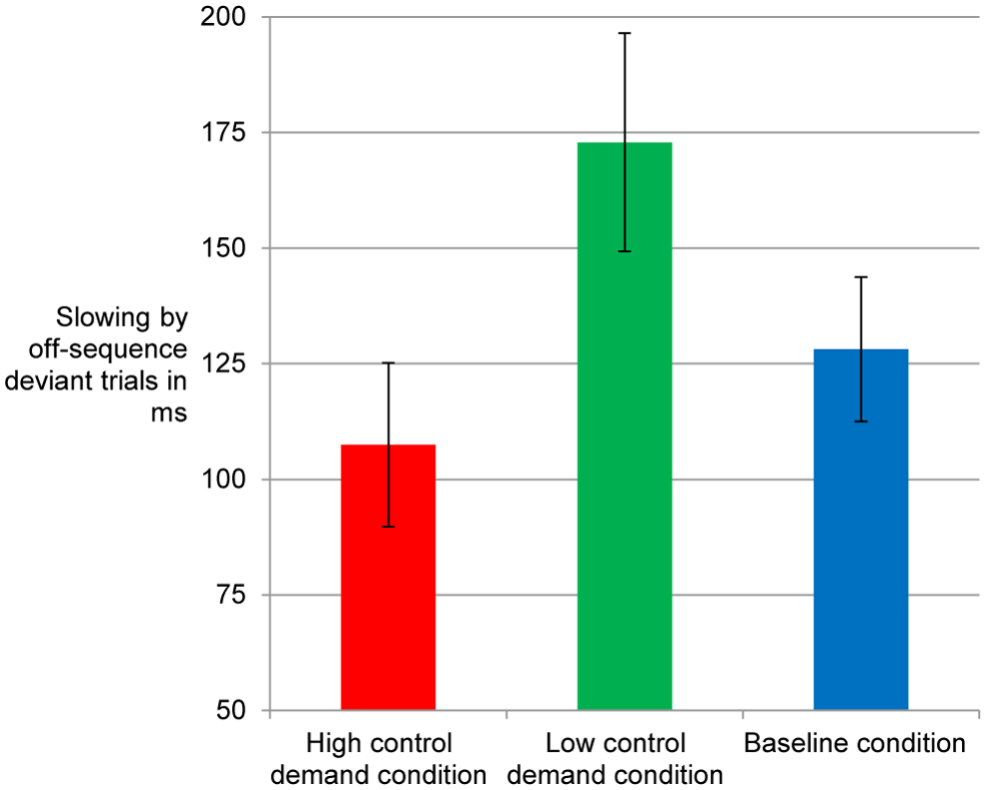
**Reaction time slowing in trials with off-sequence deviants in the SRT.** Error bars: between-subjects standard error of the mean.

In line with other implicit and incidental sequence learning studies we assessed sequence knowledge indirectly by comparing trials that follow the fixed repeating sequence with off-sequence trials at the end of practice. The RT difference between regular and deviant (plus following) trials in Block 7 is displayed in **Figure 3**. The ANOVA with control demand condition as between subjects factor showed a main effect of control demand, *F*(2,97) = 3.33, MSE = 11539.79, *p* = 0.04, $\eta_{p}^{2}$ = 0.064. Slowing was strongest for participants in the low control demand condition and weakest for those of the high control demand condition (these conditions yielded the only significant pair comparison according to Tukey-HSD, *p* = 0.04). The baseline condition lay in between.

### SEQUENCE FOLLOWED IN AMBIGUOUS TRIALS

**FIGURE 4:**
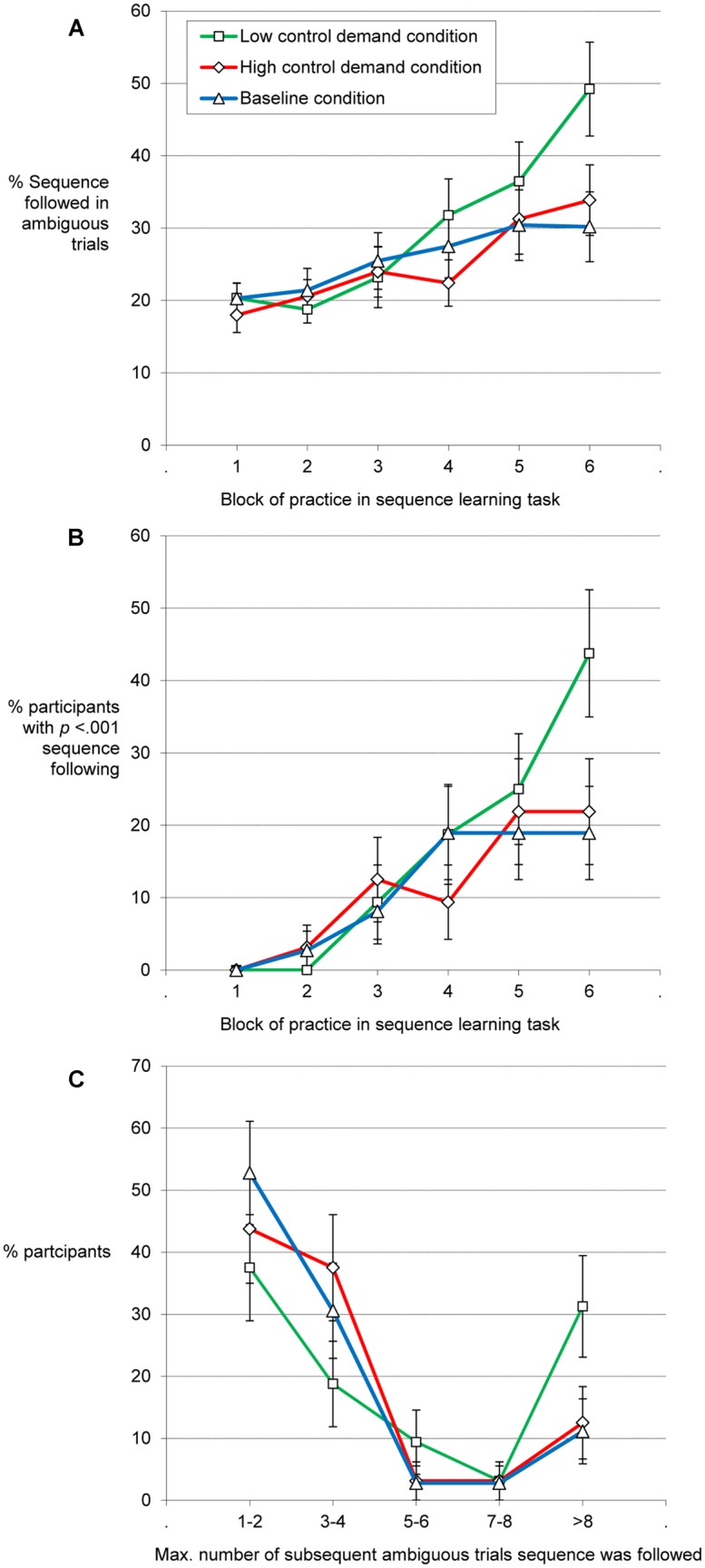
**The average rate of % ambiguous trials the sequence was followed in the SRT increases over blocks of practice (chance level = 16.7%; A). (B)** Shows the proportion of participants using the sequence in at least 50% of the ambiguous trials of the respective block (i.e., *p* < 0.001 for that participant in that block). **(C)** Shows the distribution of sequence usage in the SRT. A larger proportion of participants of the low control demand condition as compared to the other conditions showed long chains of consistent sequence memory-based responses on randomly interspersed ambiguous trials. Error bars: between-subjects standard error of the mean or the proportion.

**Figure 4A** suggests a practice-related increase in this dependent measure – the rate of ambiguous stimuli eliciting a response according to the repeating sequence. The mixed ANOVA with the factors block of practice and control demand condition showed a main effect of block of practice, *F*(3.48,337.48) = 15.78, MSE = 444.96, *p* < 0.001, $\eta_{p}^{2}$ = 0.14, and an interaction of practice and control demand condition, *F*(6.96,337.48) = 2.25, MSE = 444.96, *p* = 0.038, $\eta_{p}^{2}$ = 0.04, but no main effect of control demand condition, *F*(2,97) = 1.1. The increase in sequence following across blocks was strongest in the low control demand condition. As detailed below, between-participant variability in sequence-following in ambiguous trials was substantial. Therefore, we secured that the abovementioned pattern of results also holds with a more robust statistic. For this we determined the percentage of participants per condition and block of practice who showed above chance sequence following. We determined (based on the binomial distribution) how many sequence following responses within the 12 ambiguous trials per block of practice a participant should accumulate to be classified as an above-chance sequence follower for that block. Seven of 12 responses (i.e., >50% sequence following) are sufficient for *p* < 0.001. Supporting the above analysis, the percentage of sequence followers (**Figure 4B**) showed a similar pattern as the average rate of sequence following (**Figure 4A**). It increased the most in the low control demand condition, *X*^2^ (2) = 6.93, *p* = 0.031, for the across-condition comparison of the rate in the last block of practice. Note that the Block 6 rate also mirrors the overall increase with practice, as all conditions started from 0 in Block 1.

Several participants eventually started to consistently respond to the randomly interspersed ambiguous trials according to what the fixed sequence would have suggested. Run analyses were employed to explore the consistency of sequence following. Guessing should lead to sequence-followed responses on individual ambiguous trials, but not on whole runs of them. Consistent replacement of random key presses to ambiguous stimuli by sequence memory-based responses was captured by determining the maximum run length of sequence-following responses in ambiguous trials. We used the ambiguous trials as probes of sequence following that were randomly inserted into the repeating sequence of regular trials. Thus, runs span over many regular trials. For instance, a participant with a maximum run length of 30 has responded according to the fixed sequence without interruption for more than two blocks of practice (i.e., 12 ambiguous trials per block).

Cases where participants started to consistently respond according to the repeating sequence were especially pronounced in the low control demand condition. The maximum run length of sequence-consistent responses on subsequent ambiguous trials determined per participant was on average *M* = 8.9. It was *M* = 4.6 in the high control demand and the baseline condition. As depicted in **Figure 4C**, the distribution was heavily skewed in all conditions, as many participants did not show consistent usage of sequence knowledge in ambiguous trials. Yet, the low control demand condition yielded a high proportion of participants with especially long runs as compared to the other conditions. While 14 of the participants of the low control demand condition showed runs longer than four (four being the median of this condition; *p* < 0.001 for four consecutive hits; *Maximum* = 54 ambiguous trials), only six of the participants in both the high control demand condition and the baseline condition (*Maximum* = 30 and 29) showed sequence-consistent responses of the same run-length, *X*^2^(2) = 7.74, *p* = 0.025. In summary, different indicators converge in suggesting stronger usage of incidentally acquired sequence knowledge following the low control demand condition compared to the high control demand condition (and intermediate performance for the baseline condition).

### FOLLOW-UP ANALYSES ON ERROR RATES AND REACTION TIMES

**FIGURE 5:**
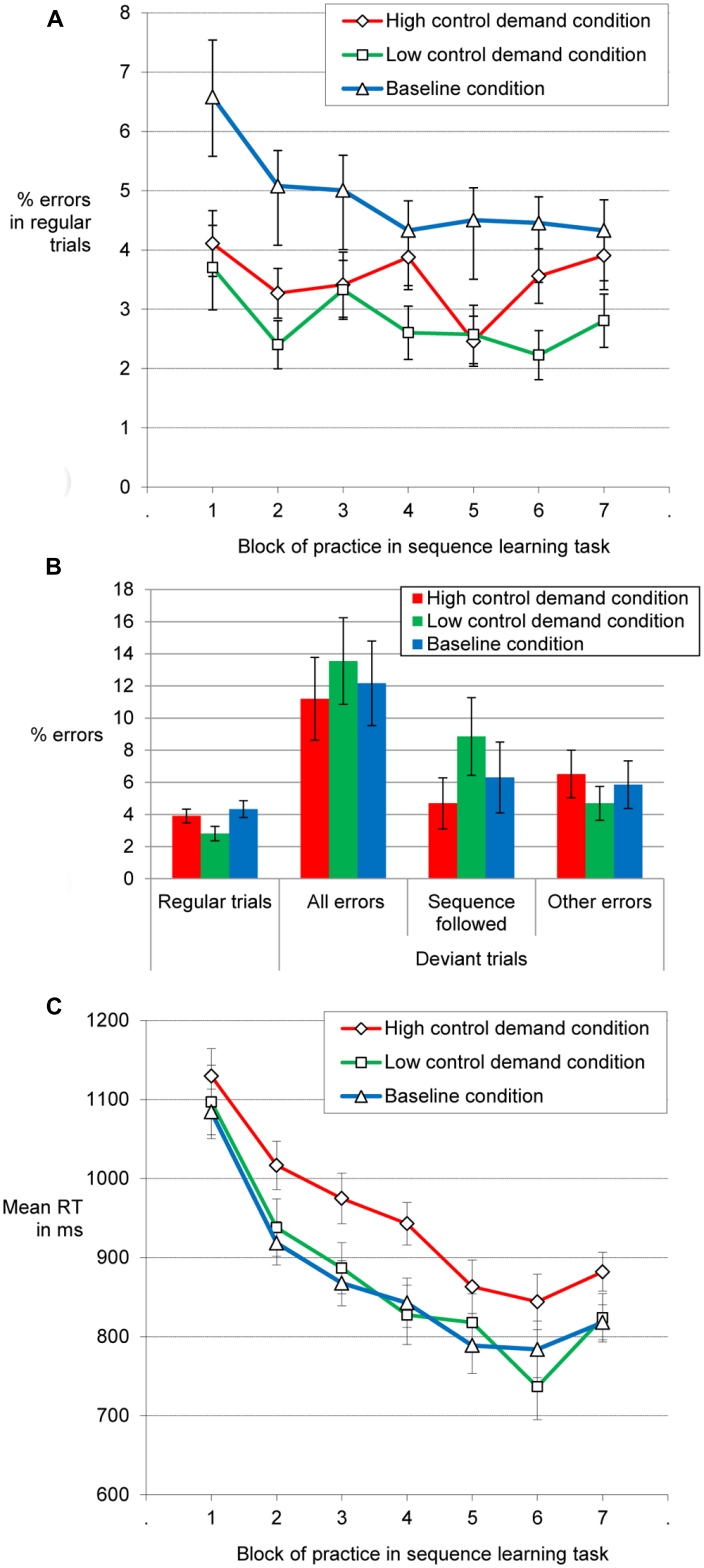
**Error rates in regular trials over blocks of practice **(A)**, error rates and error type in deviant and regular trials of Block 7 **(B)**, and reaction times in correct regular trials over the course of practice **(C)**.** Error bars: between-subjects standard error of the mean.

Unexpectedly, the mean error rate for the regular trials of Blocks 1 to 6 of the SRT (**Figure 5A**) was higher for the baseline condition (*M* = 5%) compared to the low control demand condition (*M* = 2.8%) and the high control demand condition (*M* = 3.4%). The baseline condition differed from the other two conditions according to Tukey-HSD (*p*s < 0.05). An ANOVA including block of practice and control demand condition showed a main effect of practice, as error rates decreased, *F*(3.27,316.744) = 5.08, MSE = 9.19, *p* = 0.001, $\eta_{p}^{2}$ = 0.05, and a main effect of control demand condition, *F*(2,97) = 8.16, MSE = 31.93, *p* < 0.001, $\eta_{p}^{2}$ = 0.14. There was no interaction of block and condition (*F* = 1.08).

An analysis of the error rates in Block 7 (**Figure 5B**) showed that participants produced more errors in deviant trials (compared to regular trials). Error rate increased when exclusively taking into account errors in line with the disrupted sequence, but also when only considering errors in which participants neither followed the sequence nor the current off-sequence stimulus. An ANOVA including the error rates in regular vs. in deviant trials resulted in a main effect of trial type, *F*(1,97) = 32.31, MSE = 112.2, *p* < 0.001, $\eta_{p}^{2}$ = 0.25. There was neither a main effect of nor an interaction involving control demand condition (*F*s < 1). A large proportion of errors in deviant trials were responses in line with what the repeating sequence would have suggested. A main effect of trial type (but no effects involving control demand condition) was also obtained, when comparing error rate on regular trials with the rate of sequence following in deviant trials, *F*(1,97) = 5.23, MSE = 73.82, *p* = 0.024, $\eta_{p}^{2}$ = 0.05. The pattern of a higher error rate in deviant as compared to regular trials also held when only considering errors that were not in line with the response suggested by the repeating sequence, *F*(1,97) = 7.18, MSE = 29.98, *p* = 0.009, $\eta_{p}^{2}$ = 0.07.

Last we analyzed how RT developed across blocks of practice in regular trials. While **Figure 5C** suggests that performance on regular trials was slowest in the high control demand condition, this was not confirmed by an ANOVA with block of practice and control demand condition as factors. We obtained a main effect of block of practice, reflecting that participants became faster over the six blocks of practice, *F*(2.38,230.94) = 132.56, MSE = 19705.33, *p* < 0.001, $\eta_{p}^{2}$ = 0.58. However, there was neither a main effect of control demand condition, *F*(2,97) = 2.06, nor an interaction of block and control demand condition, *F*(4.76,230.94) = 1.59. Note that an ANOVA involving trial type (regular trials vs. ambiguous trials) and block of practice did not show a main effect or interaction involving control demand condition either (*F*s < 1).

### VERBALIZABLE SEQUENCE KNOWLEDGE

The three experimental conditions did not differ with respect to the frequency with which the matches between verbalized sequence parts and practiced sequence were obtained by random matching in the simulation. The average relative frequencies were 13.39, 14.27, and 15.01%, for the low and high control demand condition and the baseline condition respectively (*F* < 1). As we administered the interview after the test block containing deviant trials, one could suspect that the measure of verbalizable sequence knowledge is too noisy to be useful. However, we obtained significant Spearman rank correlations of the measure with RT slowing on deviant trials (*r* = -0.395) and with the proportion of ambiguous trials responded to according to the fixed sequence in the last block of practice (*r* = -0.501; *p*s < 0.001). Thus, participants showing stronger behavioral signs of sequence knowledge also verbalized sequence patterns that were less frequently obtain in a random matching simulation (i.e., their verbalization was less likely based on guessing).

## DISCUSSION

We observed transfer between two incidental learning tasks, the alphabet verification task and the SRT. Participants who had the opportunity to discover *and* apply (low control demand condition) a shortcut in the first task, were more likely to apply a different shortcut in the second incidental learning task compared to participants in the high control demand condition. Low demands to adhere to instruction-based task processing in the alphabet verification task (i.e., option to skip to check some string positions without that this would lead to errors) apparently were transferred to the SRT (i.e., respond based on sequence memory rather than based on stimuli). Less usage of sequence knowledge was observed in the high control demand condition. These participants had experienced that instruction-coherent task processing has to be maintained as a shortcut would lead to errors in the alphabet verification task. The participants of the baseline condition showed intermediate application of sequence knowledge.

The two incidental learning tasks employed were highly dissimilar in terms of stimuli, responses, and hidden regularity that could be exploited for task processing. Thus, the transfer across tasks rules out that stimulus-specific processing episodes rather than learning of control demands can account for the results. Rather, the experiment illustrates general demand effects – an issue important and hard to control in research with human participants. Hertwig and Ortmann (2001) have for instance suggested that research participants in psychological experiments often search for hidden regularities in the task material, because they suspect that task instructions convey a misleading or incomplete picture of what the experiment is really about (see also Harlow, 1949; Gaissmaier and Schooler, 2008). After taking part in an incidental learning experiment, research participants might (often falsely) assume that hidden task regularities might be waiting to be found and safe to exploit in other experiments of the same or maybe even other research labs. This might distract them from performing tasks as instructed, threatening the validity of studies not interested in incidental learning and instruction following.

As the task material of the low control demand condition was set up to support the belief that exploitable task regularities might exist, participants might have been inclined to also search and apply shortcuts in the SRT afterward. Crucially, participants in the low control demand condition experienced no costs (i.e., errors) in applying the shortcut (rather than processing the alphanumeric strings as instructed). The baseline condition tended to be more similar to the high control demand condition than to the low control demand condition. This would suggest a larger impact of experiencing the *lack* of the demand to control shortcut usage on performance in a subsequent incidental learning task (rather than experiencing the demand to continue instruction-coherent task processing). This might seem plausible if the demand to follow instructions is default and rewarded in everyday life (cf. Hayes et al., 1986, 2001; Törneke et al., 2008). Currently we cannot distinguish these variants as only the difference between the low and the high control demand condition was statistically robust.

The current study at least provides tentative evidence for distinguishing influences of control demands on applying shortcut options from influences on learning about these shortcut options in the first place (cf. ErEl and Meiran, 2011). In principle, participants in the low control demand condition might either have been better at learning about the fixed repeating sequence, better at applying it, once they have learned about it, or both. Our measure of verbalizable sequence knowledge did not differ between the control demand conditions (though it correlated with performance indicators, suggesting that it was sensitive). This suggests that the control demand conditions differed primarily in applying rather than in knowing the fixed repeating sequence in the SRT.

The finding of transfer between incidental learning tasks is remarkable given that researchers have struggled to obtain transfer between structurally equivalent thought problems (cf. Helfenstein and Saariluoma, 2006; Frensch and Haider, 2008; but see Green et al., 2010). In the current study participants seemed to transfer the knowledge that shortcut options might exist and can be safely exploited to a different incidental learning task presented subsequently. Verbal reports suggest that this knowledge was explicit. Currently we can only speculate on the role of verbal knowledge in transfer between incidental learning tasks as data for direct comparisons of transfer in incidental (i.e., with verbal knowledge) vs. implicit learning tasks (i.e., without verbal knowledge) are lacking. Note however that according to implicit learning studies at least some transfer seems to be possible even without verbal knowledge of the task regularity. For instance, Leber et al. (2009) reported that participants who have adopted one attentional set (feature search mode vs. singleton detection mode) in training transferred it to another session despite changes in the coloring of the search targets. Turk-Browne and Scholl (2009) reported that visual statistical regularities, with respect to temporal sequence, transferred to spatial sequencing and *vice versa*. Stadler et al. (2000; see also Newell and Bright, 2002) reported the transfer of implicit knowledge about defining features of number strings across formats (digits vs. words).

The finding of transfer of control demands is theoretically relevant as such task-general influences on performance imply the item-general operation of control processes. The current work might contribute to alter the perspective on cognitive control in strategy change. Some models of skill acquisition focus on the aspect that strategy change can help to overcome attention-demanding task processing by applying a (memory-based) shortcut (e.g., Logan, 1988, 1992). However, in his instance theory of automatization, strategy change (e.g., from calculating simple arithmetic problems to retrieving the answer from memory), is a mandatory consequence of task practice. As soon as the memory strength is sufficient, the shortcut is automatically applied. Importantly, transfer across tasks with different types of shortcuts operating on different types of task material is not to be expected according to the instance theory and related models of strategy change in skill acquisition (e.g., Cousineau and Larochelle, 2004), because shortcuts are based on knowledge that has to be acquired individually for each stimulus (e.g., the correct solution to an arithmetic problem). According to this perspective, strategy change relying on *automatic* memory retrieval of answers to formerly presented problems can free attentional resources. There is neither room for transfer across incidental learning tasks, nor for control processes that might modulate whether or not shortcut knowledge is applied. This changes, if participants can decide to apply or not apply a shortcut option which they have incidentally learned (e.g., Touron and Hertzog, 2004a,b; Gaschler and Frensch, 2009). According to the learning-plus-decision perspective on strategy change in incidental learning, incidentally learning about a shortcut option could lead to a demand of cognitive control — namely when shortcut application leads to errors. The current study provides first evidence for that such control demands might transfer across incidental learning tasks. It extends recent work showing that learning processes involved in strategy change can generalize across specific stimuli within a task: Strategy change is not confined to learning a shortcut specifically for each stimulus (cf. Logan, 1988, 1992), but instead transfers across stimuli within a task (cf. Gaschler et al., 2014a). For instance, Wilkins and Rawson (2010, p. 1134) conceptualized item-general practice gains as performance improvements “that accrue to all stimulus tokens of a given type, including both practiced and novel tokens of that type.” The current work suggests that this might even include different tasks.

Apart from the learning-plus-decision perspective on strategy change discussed above, there is another theoretical perspective that can account for transfer between incidental learning tasks, but makes different predictions for the specific pattern of transfer that should occur: theories that places emphasis on potential psychological resources needed to refrain from shortcut usage. According to research on ego depletion (e.g., Baumeister et al., 2007; Hagger et al., 2010), working on a demanding task can exhaust a control resources that are then not available for the next task to come. Assuming that high demand conditions deplete cognitive control resources more than low demand conditions would have led to the following prediction: Working on a task that demands to refrain from using a shortcut option should have led to more shortcut usage in a later incidental learning task compared to a condition in which participants did not have to refrain from applying a shortcut in the first task. The least depletion of control resources should have taken place if participants do not have to work on a prior task at all. Thus, the baseline condition lacking experience with either variant of Task 1 should have shown the least shortcut usage. Different from these predictions, we obtained the strongest shortcut usage in the SRT in the low control demand condition. The demand to refrain from using a shortcut option in Task 1 should have diminished the capability to secure adherence to instructions in Task 2. As the alphabet verification task is tedious even when a part of the material can be skipped, the low control demand condition should have shown an intermediate level of shortcut usage, while it should have been lowest in the baseline condition (i.e., SRT only, hence least depleted). As we did not include independent measures of depletion (e.g., a pre–post-test comparison of self-reported fatigue) we are cautious to over-interpret our results with respect to resource-theories of self-control. Note that the unexpected high error rate on regular trials in the baseline condition is at odds with the resource perspective as well – rather predicting a lower error rate in the least depleting condition. Instead, our results are in line with theories that conceptualize strategy change in incidental learning tasks as a phenomenon involving (a) learning of the task regularity and (b) a decision to apply or not apply the shortcut (e.g., Strayer and Kramer, 1994; Touron and Hertzog, 2004a,b; Haider and Frensch, 2005; Haider et al., 2005).

While past work has documented that shortcut application can take place in an all-or-none manner, generalizing even to novel stimuli within a task (e.g., Gaschler and Frensch, 2007; Gaschler et al., 2014a), the current work, in addition, presents first evidence for transfer across different incidental learning tasks. Distinguishing between influences of specific processing episodes versus adaptation to general task structure is a key-problem shared by the literature on conflict adaptation (e.g., Jacoby et al., 2003; Mayr et al., 2003; Ullsperger et al., 2005; Schmidt and Besner, 2008) and the literature on strategy change in skill acquisition. One research strategy to accumulate evidence for adaptation to control demands (rather than to specific processing episodes with specific stimuli) has been the study of transfer of control from one task to another in task switching (e.g., Egner, 2008; Fernandez-Duque and Knight, 2008; but see Rünger et al., 2010). In that work the focus is on transfer of control between tasks on a trial-by-trial basis. Others, have focused on learning of parameters controlling strategy selection over many trials (e.g., Gray et al., 2006; Gaschler and Frensch, 2009; Schouppe et al., 2014). Models of strategy selection (e.g., Rieskamp and Otto, 2006; Marewski and Schooler, 2011) might be expanded such that they can capture task-general learning of applicability vs. non-applicability of shortcuts.

Last we would like to highlight that it is interesting to consider alternative task orders to study transfer across incidental learning tasks. Transfer of control demands across incidental learning tasks could be studied in either direction taking the alphabet verification task or the SRT as independent and dependent variable or *vice versa*. Control demands could be manipulated in the SRT by varying the amount of randomly inserted stimuli breaking the repeating sequence (cf. Verwey and Wright, 2014). However, for issues tied to task difficulty, we decided to use performance in the alphabet verification task as the independent variable in our experiment, varying whether participants (a) could safely apply a shortcut option (b) should not apply the shortcut or (c) did not have to work on this task at all. While the SRT is instructed as a simple choice reaction task and can be solved at reaction time levels of one second or less per trial – even without applying a shortcut – the alphabet verification task is much more tedious. Reaction times rarely reduce below three seconds per trial. From the perspective of the ego depletion theory, this task should exhaust more control resources when participants have to refrain from applying a shortcut and less, when control demands are low. Yet, even when participants can safely use the shortcut and skip to check some positions of the alphanumeric strings, each trial still contains a substantial amount of string positions to be checked. Thus, even the low control demand condition should be affected by exhaustion of control resources and thus show more shortcut usage in Task 2 as compared to the baseline condition.

In the current setup we tested whether being able to use vs. having to forego using a shortcut option in a demanding task affects shortcut usage in second incidental learning task. Reversing the order of the incidental learning tasks in future studies could additionally challenge control resource accounts. Assuming that the SRT is comparatively less demanding, withholding shortcut usage in the SRT should not lead to a substantial exhaustion of control resources. From this perspective, shortcut usage in the alphabet verification task should not differ depending on prior applicability of sequence knowledge in the SRT. The learning-plus-decision perspective on strategy change however suggests that providing participants with the opportunity to find and apply a shortcut in a relatively easy task could strengthen shortcut usage in a more difficult task provided later on. Experiments on shortcut usage in arithmetic (Godau et al., 2014) indeed reflect that that offering an easy-to-find shortcut option can increase later shortcut usage. Therefore, the sequential regularity in the SRT (which can be detected and applied rather easily) could foster later shortcut usage in the alphabet verification task, if the tasks would be applied in the reversed order.

In summary, the present study offers a cognitive control perspective on strategy change in incidental learning tasks. In line with theories conceptualizing strategy change in incidental learning as a learning-plus-decision phenomenon, we observed transfer of control demands across incidental learning tasks. This provides further evidence for that control processes can be distinguished from adaptation to the specific material practiced.

## Conflict of Interest Statement

The authors declare that the research was conducted in the absence of any commercial or financial relationships that could be construed as a potential conflict of interest.

## References

[B1] Abrahamse E. L. Jiménez J. Verwey W. B. Clegg B. A. (2010). Representing serial action and perception. *Psychon. Bull. Rev.* 17 603–623 10.3758/PBR.17.5.603 21037157

[B2] Baumeister R. F. Vohs K. D. Tice D. M. (2007). The strength model of self-control. *Curr. Dir. Psychol. Sci.* 16 351–355 10.1111/j.1467-8721.2007.00534.x

[B3] Cousineau D. Larochelle S. (2004). Visual-memory search: an integrative perspective. *Psychol. Res.* 69 77–105 10.1007/s00426-003-0170-5 14986138

[B4] Duthoo W. Wühr P. Notebaert W. (2013). The hot-hand fallacy in cognitive control: repetition expectancy modulates the congruency sequence effect. *Psychon. Bull. Rev.* 20 798–805 10.3758/s13423-013-0390-7 23371807

[B5] Egner T. (2008). Multiple conflict-driven control mechanisms in the human brain. *Trends Cogn. Sci.* 12 376–380 10.1016/j.tics.2008.07.001 18760657

[B6] ErEl H. Meiran N. (2011). Mindset changes lead to drastic impairments in rule finding. *Cognition* 119 149–165 10.1016/j.cognition.2011.01.002 21316041

[B7] Fernandez-Duque D. Knight M. (2008). Cognitive control: dynamic, sustained, and voluntary influences. *J. Exp. Psychol. Hum. Percept. Perform.* 34 340–355 10.1037/0096-1523.34.2.340 18377175

[B8] Frensch P. A. Haider H. (2008). “Transfer and expertise,” in *Learning and Memory – A Comprehensive Reference* Vol. 2 ed. Roediger H. L. III (Oxford: Elsevier), 579–596 10.1016/B978-012370509-9.00177-7

[B9] Gaissmaier W. Schooler L. J. (2008). The smart potential behind probability matching. *Cognition* 109 416–422 10.1016/j.cognition.2008.09.007 19019351

[B10] Gaschler R. Frensch P. A. (2007). Is information reduction an item-specific or an item-general process? *Int. J. Psychol.* 42 218–228 10.1080/00207590701396526

[B11] Gaschler R. Frensch P. A. (2009). When vaccinating against information reduction works and when it does not work. *Psychol. Stud.* 54 42–53 10.1007/s12646-009-0006-5

[B12] Gaschler R. Frensch P. A. Cohen A. Wenke D. (2012). Implicit sequence learning based on instructed task set. *J. Exp. Psychol. Learn. Mem. Cogn.* 38 1389–1407 10.1037/a0028071 22545612

[B13] Gaschler R. Marewski J. N. Frensch P. A. (2014a). Once and for - all How people change strategy to ignore irrelevant information in visual tasks. *Q. J. Exp. Psychol.* 10.1080/17470218.2014.961933 [Epub ahead of print]. 25203902

[B14] Gaschler R. Schwager S. Umbach V. J. Frensch P. A. Schubert T. (2014b). Expectation mismatch: differences between self-generated and cue-induced expectations. *Neurosci. Biobehav. Rev.* 46 139–157 10.1016/j.neubiorev.2014.06.009 24971824

[B15] Godau C. Haider H. Hansen S. Schubert T. Gaschler R. (2014). Spontaneously spotting and applying shortcuts in arithmetic – a primary school perspective on expertise. *Front. Psychol.* 5:556 10.3389/fpsyg.2014.00556 PMC4051128 24959156

[B16] Gray W. D. Sims C. R. Fu W. T. Schoelles M. J. (2006). The soft constraint hypothesis: a rational analysis approach to resource allocation for interactive behavior. *Psychol. Rev.* 113 461–482. 16802878 10.1037/0033-295X.113.3.461

[B17] Green A. Wright M. (2003). Reduction of task-relevant information in skill acquisition. *Eur. J. Cogn. Psychol.* 15 267–291.

[B18] Green C. S. Pouget A. Bavelier D. (2010). Improved probabilistic inference as a general learning mechanism with action video games. *Curr. Biol.* 23 1573–1579 10.1016/j.cub.2010.07.040 20833324 PMC2956114

[B19] Hagger M. S. Wood C. Stiff C. Chatzisarantis N. L. (2010). Ego depletion and the strength model of self-control: a meta-analysis. *Psychol. Bull.* 136 495–525 10.1037/a0019486 20565167

[B20] Haider H. Frensch P. A. (1996). The role of information reduction in skill acquisition. *Cogn. Psychol.* 30 304–337 10.1006/cogp.1996.0009 8660787

[B21] Haider H. Frensch P. A. (1999). Information reduction during skill acquisition: the influence of task instruction. *J. Exp. Psychol. Appl.* 5 129–151.

[B22] Haider H. Frensch P. A. (2002). Why aggregated learning follows the power law of practice when individual learning does not: comment on Rickard (1997, 1999), Delaney etal. (1998), and Palmeri (1999) *J. Exp. Psychol. Learn. Mem. Cogn.* 28 392–406 10.1037/0278-7393.28.2.392 11911396

[B23] Haider H. Frensch P. A. (2005). The generation of conscious awareness in an incidental learning situation. *Psychol. Res.* 69 399–411. 15944861 10.1007/s00426-004-0209-2

[B24] Haider H. Frensch P. A. Joram D. (2005). Are strategy shifts caused by data-driven processes or by voluntary processes? *Conscious. Cogn.* 14 495–519 10.1016/j.concog.2004.12.002 16091268

[B25] Haider H. Rose M. (2007). How to investigate insight: a proposal. *Methods* 42 49–58. 17434415 10.1016/j.ymeth.2006.12.004

[B26] Harlow H. F. (1949). The formation of learning sets. *Psychol. Rev.* 56 51–65 10.1037/h0062474 18124807

[B27] Hayes S. C. Barnes-Holmes D. Roche B. (eds) (2001). *Relational Frame Theory: A Post-Skinnerian Account of Human Language and Cognition*. New York: Kluwer Academic/Plenum Publishers. 10.1016/s0065-2407(02)80063-5 11605362

[B28] Hayes S. C. Brownstein A. J. Zettle R. D. Rosenfarb I. Korn Z. (1986). Rule-governed behavior and sensitivity to changing consequences of responding. *J. Exp. Anal. Behav.* 45 237–256 10.1901/jeab.1986.45-237 16812448 PMC1348236

[B29] Helfenstein S. Saariluoma P. (2006). Mental contents in transfer. *Psychol. Res.* 70 293–303 10.1007/s00426-005-0214-0 16001278

[B30] Hertwig R. Ortmann A. (2001). Experimental practices in economics: a methodological challenge for psychologists? *Behav. Brain Sci.* 24 383–451. 11682798 10.1037/e683322011-032

[B31] Hoyndorf A. Haider H. (2009). The “Not Letting Go” phenomenon: accuracy instructions can impair behavioral and metacognitive effects of implicit learning processes. *Psychol. Res.* 73 695–706 10.1007/s00426-008-0180-4 18998161

[B32] Jacoby L. L. Lindsay D. S. Hessels S. (2003). Item-specific control of automatic processes: stroop process dissociations. *Psychon. Bull. Rev.* 10 634–644 10.3758/BF03196526 14620358

[B33] Leber A. B. Kawahara J.-I. Gabari Y. (2009). Long-term abstract learning of attentional set. *J. Exp. Psychol. Hum. Percept. Perform.* 35 1385–1397 10.1037/a0016470 19803644 PMC4654949

[B34] Logan G. D. (1988). Toward an instance theory of automatization. *Psychol. Rev.* 95 492–527 10.1037/0033-295X.95.4.492

[B35] Logan G. D. (1992). Shapes of reaction-time distributions and shapes of learning curves: a test of the instance theory of automaticity. *J. Exp. Psychol. Learn. Mem. Cogn.* 18 883–914 10.1037/0278-7393.18.5.883 1402715

[B36] Marewski J. N. Schooler L. J. (2011). Cognitive niches: an ecological model of strategy selection. *Psychol. Rev.* 118 393–437 10.1037/a0024143 21744978

[B37] Mayr U. Awh E. Laurey P. (2003). Does conflict adaptation require executive control? *Nat. Neurosci.* 6 450–452. 12704394 10.1038/nn1051

[B38] Newell B. R. Bright J. E. H. (2002). Evidence against hyperspecificity in implicit invariant learning. *Q. J. Exp. Psychol.* 55A, 1109–1126 10.1080/02724980244000062 12420987

[B39] Niessen C. Eyferth K. Bierwagen T. (1999). Modeling cognitive processes of experienced air traffic controllers. *Ergonomics* 42 1507–1520 10.1080/001401399184857 10582037

[B40] Nissen M. J. Bullemer P. (1987). Attentional requirements of learning: evidence from performance measures. *Cogn. Psychol.* 19 1–32 10.1016/0010-0285(87)90002-8

[B41] Reason J. (1990). *Human Error.* New York: Cambridge University Press 10.1017/CBO9781139062367

[B42] Rieskamp J. Otto P. E. (2006). SSL: a theory of how people learn to select strategies. *J. Exp. Psychol. Gen.* 135 207–236 10.1037/0096-3445.135.2.207 16719651

[B43] Ridderinkhof K. R. Ullsperger M. Crone E. A. Nieuwenhuis S. (2004). The role of the medial frontal cortex in cognitive control. *Science* 306 443–447 10.1126/science.1100301 15486290

[B44] Rünger D. Frensch P. A. (2008). How incidental sequence learning creates reportable knowledge: the role of unexpected events. *J. Exp. Psychol. Learn. Mem. Cogn.* 34 1011–1026 10.1037/a0012942 18763888

[B45] Rünger D. Schwager S. Frensch P. A. (2010). Anticipatory cognitive control: no evidence for cross-task modulation. *J. Exp. Psychol. Hum. Percept. Perform.* 36 136–146 10.1037/a0017172 20121300

[B46] Schmidt J. R. Besner D. (2008). The Stroop effect: why proportion congruent has nothing to do with congruency and everything to do with contingency. *J. Exp. Psychol. Learn. Mem. Cogn.* 34 514–523 10.1037/0278-7393.34.3.514 18444752

[B47] Schouppe N. de Ferrerre E. Van Opstal F. Braem S. Notebaert W. (2014). Conscious and unconscious context-specific cognitive control. *Front. Psychol.* 5:539 10.3389/fpsyg.2014.00539 PMC4045158 24926275

[B48] Schvaneveldt R. W. Gomez R. L. (1998). Attention and probabilistic sequence learning. *Psychol. Res.* 61 175–190 10.1007/s004260050023

[B49] Schwager S. Rünger R. Gaschler R. Frensch P. A. (2012). Data-driven sequence learning or search – what are the prerequisites for the generation of explicit sequence knowledge? *Adv. Cogn. Psychol.* 8 132–143 10.5709/acp-0110-4 22723812 PMC3376888

[B50] Shanks D. R. Wilkinson L. Channon S. (2003). Relationship between priming and recognition in deterministic and probabilistic sequence learning. *J. Exp. Psychol. Learn. Mem. Cogn.* 29 248–261 10.1037/0278-7393.29.2.248 12696813

[B51] Stadler M. A. Warren J. L. Lesch S. L. (2000). Is there cross-format transfer in implicit invariance learning? *Q. J. Exp. Psychol.* 52A, 235–245 10.1080/713755879 10718072

[B52] Strayer D. L. Kramer A. F. (1994). Strategies and automaticity: I. Basic findings and conceptual framework. *J. Exp. Psychol. Learn. Mem. Cogn.* 20 318–341 10.1037/0278-7393.20.2.318

[B53] Törneke N. Luciano C. Valdivia Salas S. (2008). Rule-governed behavior and psychological problems. *Int. J. Psychol. Psychol. Ther.* 8 141–156.

[B54] Touron D. R. Hertzog C. (2004a). Distinguishing age differences in knowledge, strategy use, and confidence during strategic skill acquisition. *Psychol. Aging* 19 452–466 10.1037/0882-7974.19.3.452 15382996

[B55] Touron D. R. Hertzog C. (2004b). Strategy shift affordance and strategy choice in young and older adults. *Mem. Cogn.* 32 298–310 10.3758/BF03196860 15190721

[B56] Tubau E. Hommel B. López-Moliner J. (2007). Modes of executive control in sequence learning: from stimulus-based to plan-based control. *J. Exp. Psychol. Gen.* 136 43–63 10.1037/0096-3445.136.1.43 17324084

[B57] Turk-Browne N. B. Scholl B. J. (2009). Flexible visual statistical learning: transfer across space and time. *J. Exp. Psychol. Hum. Percept. Perform.* 35 195–202 10.1037/0096-1523.35.1.195 19170482

[B58] Ullsperger M. Von Cramon Y. Bylsma L. Botvinick M. (2005). The conflict adaptation effect: it’s not just priming. *Cogn. Affect. Behav. Neurosci.* 5 467–472 10.3758/CABN.5.4.467 16541815

[B59] Underwood G. Crundall D. Chapman P. (2002). Selective searching while driving: the role of experience in hazard detection and general surveillance. *Ergonomics* 45 1–12 10.1080/00140130110110610 11964191

[B60] Verwey W. B. Wright D. L. (2014). Learning a keying sequence you never executed: evidence for independent, concurrent associative and motor chunk learning. *Acta Psychol.* 151 24–31 10.1016/j.actpsy.2014.05.017 24929277

[B61] Wilkins N. J. Rawson K. A. (2010). Loss of cognitive skill across delays: constraints for theories of cognitive skill acquisition. *J. Exp. Psychol. Learn. Mem. Cogn.* 36 1134–1149 10.1037/a0019998 20804290

